# Comparison between two methods of the immediate post-placental insertion of copper intrauterine device in vaginal birth—a protocol for a randomized clinical trial

**DOI:** 10.1186/s13063-022-07041-x

**Published:** 2022-12-27

**Authors:** Thuany Bento Herculano, Fernanda Garanhani Surita, Cássia Raquel Teatin Juliato, Patrícia Moretti Rehder

**Affiliations:** Department of Obstetrics and Gynecology, School of Medical Science, University of Campinas, Av. Alexander Fleming, Campinas, SP 101 Brazil

**Keywords:** Intrauterine device (IUD), Contraception, Postpartum period, Vaginal birth, Immediate post-placental insertion

## Abstract

**Background:**

Ensuring effective and long-term contraception in the immediate postpartum period is an effective strategy for reducing unplanned pregnancies. In the meantime, the intrauterine device (IUD) is an excellent option. The aim of our study was to evaluate the best way to insert post-placental IUDs in the immediate postpartum period. Discomfort during insertion, expulsion rate, uterine perforation rate, and proper positioning 40–60 days postpartum will be analyzed.

**Methods:**

Randomized, controlled, open clinical trial. The study group will be composed of women between 18 and 43 years old who are admitted for vaginal birth at the Women’s Hospital of the State University of Campinas and who wish to use the IUD as a contraceptive method. The sample will be randomized into two insertion groups: manual and forceps. To calculate the sample size, the method of comparing the proportion between 2 groups was used, setting the level of significance alpha at 5% (alpha=0.05) and the power of the sample at 80% (beta=0.20). Based on the results, it was estimated that a sample of *n*=186 women (*n*=93 with manual insertion and *n*=93 with forceps) would be representative for comparison of expulsion between the 2 groups. All participants will undergo a postpartum consultation 40–60 days after birth with transvaginal ultrasound to assess the proper placement of the IUD.

**Discussion:**

Insertion of an IUD in the immediate postpartum period has been considered a good option to increase coverage and access to contraception, and its benefit outweighs the inconvenience of a higher expulsion rate.

**Trial registration:**

This study was approved by the Ethics and Research Commission of UNICAMP (CAAE: 50497321.4.0000.5404) and the Brazilian Registry of Clinical Trials (REBEC) (number RBR-4j62jv6). This is the first version of the study protocol approved on 11/12/2021 prior to the start of participant recruitment.

**Supplementary Information:**

The online version contains supplementary material available at 10.1186/s13063-022-07041-x.

## Background

The first 12 months after birth is a period when a subsequent pregnancy is at increased maternal/infant risk [1]. The importance of postpartum reproductive planning is already clearly documented, with a reduction in the risk of miscarriage, low weight at birth, neonatal and maternal death, premature delivery, and anemia [2, 3]. Reproductive planning could prevent more than 30% of maternal deaths and 10% of child deaths if the interpartum interval was at least 2 years [4].

After childbirth, 40% of women who need contraception do not have access to contraceptive methods [5]. This is aggravated in times of crisis, such as the COVID-19 pandemic, in which access to health services was restricted and reproductive planning programs were reduced, despite their essential character [6, 7].

A study of nearly 15,000 women showed that 95% of those who wanted contraception underwent insertion of an intrauterine device (IUD) during hospitalization, while only 45% of women did so after discharge [8]. For 40% of women, sexual activity returns before 6 weeks postpartum and without the use of contraceptives [9]. Thus, delaying the start of effective contraception can favor a new unplanned pregnancy in a short period of time [10].

Compared with definitive sterilization, the use of the IUD is simpler, less expensive and immediately reversible. Insertion of the IUD after delivery can avoid the discomfort related to interval insertion (≥ 4 weeks postpartum) reported by some women who undergo the procedure without anesthesia, sometimes requiring cervical dilation.

However, immediate insertion, that is, that performed within 10 min after delivery of the placenta (post-placental IUD), can also have disadvantages. The risk of expulsion is usually greater [10]. Three systematic reviews have shown that IUD insertion in the immediate postpartum period is safe and effective when compared to later insertions (more than 10 min to 48–72 h or after 6–8 weeks of delivery) [11–13]. Comparing insertion during hospitalization for delivery, immediate insertions are associated with lower expulsion rates than later insertions (e.g., 2 to 72 h after placental delivery) [11, 12]. The systematic review carried out by the Cochrane group included 9 studies. Comparing immediate insertion and after 6–8 weeks, a higher expulsion rate was found in the group with immediate insertion, but with the same proportion of women using the device at 6 months [11].

Insertion of the copper IUD after vaginal delivery can be done manually by moving the device to the uterine fundus with your fingertips or using long ring-tipped forceps, such as the modified Kelly forceps. There is also a long inserter specially developed for this purpose. However, it is not widely available on services.

The aim of our study is to evaluate the best way to insert the IUD in the immediate postpartum period: manually or with the use of modified Kelly placental forceps. Discomfort during insertion, expulsion rate, uterine perforation rate, and proper positioning 40–60 days postpartum will be analyzed.

## Methods/design

### Study type

Randomized, controlled, open clinical trial.

### Setting

The study will be conducted at the Woman’s Hospital, University of Campinas Medical School, Campinas, Brazil. The facility is a tertiary referral public hospital that offers treatment and is a referral for approximately 40 municipalities, covering a population of more than five million inhabitants.

### Outcomes

#### Primary

Expulsion rate

#### Secondary

Infection rate

Perforation rate

Discomfort during insertion

Ultrasound positioning of the IUD

Side effects (increased bleeding and dysmenorrhea)

### Inclusion and exclusion criteria

The study will include women between 18 and 43 years old, with a single pregnancy, who have vaginal birth, who wish to use the IUD as a contraceptive method, and who do not have contraindications, namely, uterine malformation; uterine fibroids that deform the cavity; diagnosis or suspicion of ovular infection; diagnosis or suspicion of infection elsewhere; active sexually transmitted infection; and severe anemia (hemoglobin less than 8.0 mg/dl during antenatal care). Women who, after signing the consent form and randomization, wish to withdraw from the study for any reason, present with fever during labor or delivery, rupture of amniotic membranes for more than 24 h, manual extraction of the placenta, postpartum hemorrhage or uterine atony will be withdrawn from the study.

### Intervention

The possibilities of postpartum contraception and the benefits of starting a method before hospital discharge will be discussed during antenatal consultations or on admission for childbirth for women with external antenatal care. Women who want the copper IUD as a contraceptive method will be informed about its durability, side effects, advantages, and disadvantages of its insertion after placental delivery. Women who wish to participate in the study will be randomly assigned to one of the groups (forceps or manual insertion), through a computer-generated numerical allocation list. Randomization will be kept confidential in a sealed envelope until the time of insertion. In the immediate postpartum period, the copper IUD will be inserted according to the result of the randomization. Only members of the research group, with specific training, will be able to insert the IUD after signing the consent form by the patient. Study participants will remain unsure to which group they were allocated.

### Follow-up

Participants will be followed on the same service. Between 40 and 60 days postpartum, they should return for postpartum consultation and pelvic ultrasound to assess the IUD and cut the wires (see Table 1). In cases where the woman has an IUD in situ and without visualization of the threads in the cervical canal, the follow-up will be exclusive with pelvic ultrasound. For women who have expelled IUDs or who have IUDs in the cervical canal, a new device of the woman’s preference will be offered. In cases where the woman is not satisfied with IUD, another contraceptive method will be offered and made available.

**Table 1:** Procedures and techniques

	Study period					
	Enrollment		Allocation	Post allocation		
	Prenatal consultations	Admission for childbirth	Randomization	Immediately after randomization	In the labor and delivery room following delivery	Postpartum consultations and pelvic ultrasound
**Timepoint**	**T−2**	**T−1**	**T0**	**T1**	**T2**	**T3**
**Enrolment:**						
**Preinclusion**	x					
**Eligible patients**		x				
**Signing of the consent form**		x				
**Allocation**			x			
**Interventions:**						
**Manual insertion**				x		
**Forceps insertion**				x		
**Assessments:**						
**Pain or discomfort during insertion**					x	
**Expulsion**						x
**Uterine perforation**						x
**Proper positioning**						x

Orientation, insertion, and removal of misplaced IUDs will be performed by a group of trained resident physicians and supervised by an experienced chief. To guarantee the standardization and constant review of the technique, periodic training offered by the researchers is planned. After insertion, the professional responsible for the procedure is recorded so that we can later correlate whether the expulsion and/or mispositioning was influenced by the experience of the professional who performed the procedure.

**Fig. 1:**
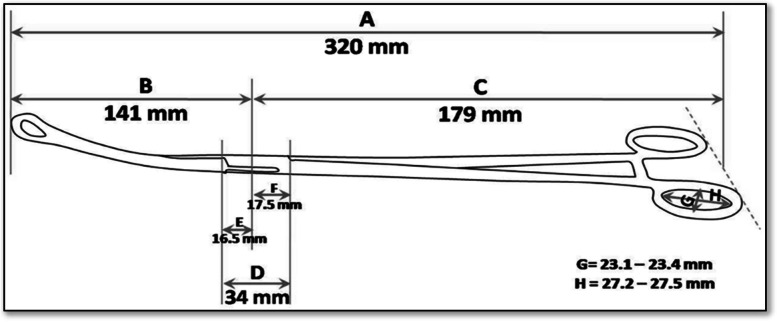
Modified Kelly forceps

The insertion technique will be briefly described below. For insertion with the forceps, the patient must be in a lithotomy position with the feet or knees supported. Asepsis of the vulva, vagina, and cervix will be performed with aqueous chlorhexidine solution. The uterus will be palpated to assess the fundus and tone. The evaluator must wear sterile gloves and must gently insert the vaginal speculum. The anterior labrum of the cervix should be clamped with a Collin clamp. The physician’s nondominant hand rested on the uterine fundus. The IUD should be secured using modified Kelly forceps (see Fig. 1). The physician's nondominant hand rested on the uterine fundus, and the IUD was gently placed through the cervix using forceps. The forceps must then be opened and withdrawn completely from the side. The clamp from the labrum of the cervix and the vaginal speculum should both then be removed.

**Fig. 2:**
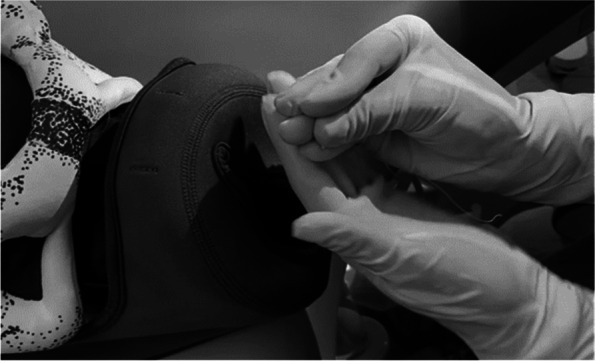
Hand position for IUD protection during insertion

For manual insertion, the patient must be in a lithotomy position with the feet or knees supported. Asepsis of the vulva, vagina, and cervix will be performed with an aqueous chlorhexidine solution. The uterus will be palpated to assess the fundus and tone. The evaluator must wear sterile gloves and must gently insert the device by holding it between the second and third fingers of the hand. The hand must be closed in order to completely protect the IUD so that it does not touch the vaginal walls (see Fig. 2). The physician’s nondominant hand rested on the uterine fundus, and the IUD was gently placed through the cervix and into the fundus.

In the puerperal review appointment, carried out between 40 and 60 days by a multidisciplinary team (physician, nurse, and psychologist), a pelvic physical examination will be performed and a questionnaire will be applied to assess, among other questions, the breastfeeding pattern (exclusive, mixed or artificial), bleeding pattern, signs of infection, pelvic pain, return to sexual activity, degree of patient satisfaction with the method, in addition to the desire to continue using the IUD or switch to other methods. When the patient is not sure whether she wants to continue with the IUD or has a transient complaint that can be clinically managed, such as increased bleeding, a new appointment will be scheduled between 30 and 60 days.

Transvaginal ultrasound will be performed at the Hospital’s Diagnostic Imaging Department to assess the proper positioning of the IUD using the Voluson 730 Expert Device, transvaginal, endocavitary probe, transvaginal preset, frequency of 6.5 MHz, mode B, with adjusted focus, gain, and depth. For normal-positioned IUDs and with satisfied patients, the wires will be cut when apparent. For badly positioned IUDs inside the uterine cavity or for those in the endocervical canal, the device will be removed, and a new device will be inserted if the patient desires. In the case of a mispositioned IUD without visible threads, removal will be performed with Hartmann forceps, when possible, or by hysteroscopy. In case of IUD expulsion, the patient will also be offered a new IUD. In the case of uterine perforation, abdominal radiography or a computed tomography scan will be performed to identify the device in the abdominal cavity. The removal will be done by laparoscopy*.* In the case of infection, the patient will be promptly treated with antibiotic therapy and the IUD will be removed if necessary*.* The data referring to the insertions will be analyzed quarterly and presented in the meeting of professors of the obstetrics department of the State University of Campinas to evaluate the feasibility of maintaining the study. In case of the need to modify the protocol of this study after evaluation by obstetrics professors, it will be communicated to the Research Ethics Committee for consideration. Participants will also be communicated at the puerperal review appointment.

Participants who have complaints related to the intrauterine device and have already attended the postpartum consultation may seek the hospital’s emergency care unit for gynecological evaluation. As many women remain in amenorrhea during lactation, when menstrual cycles return and in case of increased flow or the presence of cramps that bring discomfort, they should also return for a new gynecological evaluation.

### Sample size

To calculate the sample size, the proportion between 2 groups method for comparison was used, setting the level of significance alpha or type I error at 5% (alpha=0.05) (or 95% confidence interval) and the power of the sample at 80% (or 20% type II error (beta=0.20)), according to Hulley et al. (2007) [14], and using the expulsion ratio values after IUD insertion in vaginal delivery in each group, obtained from a literature article [15]. Based on the results, it was estimated that a sample of *n*=186 women (*n*=93 with manual insertion and *n*=93 with forceps) would be representative for comparison of expulsion between the 2 groups.

### Statistical analysis

The collected data will be coded and stored anonymously in a database created with Excel for Windows software for this purpose. Data will be allocated in tables and graphs for descriptive statistical analysis (mean, standard deviation, absolute and relative frequency distribution). The crossings involving a qualitative variable and a quantitative variable will be performed using parametric and nonparametric comparison tests, according to the distribution of data. The crossings involving two quantitative variables will be performed by calculating correlation coefficients and the crossings between two qualitative variables using association tests. Open questions will be categorized for application of statistical analysis. Continuity and discontinuation rates and their reasons will be evaluated by life table according to Kaplan–Meier. The significance level adopted for the statistical tests will be 5%. For statistical analysis, the Statistical Analysis System (SAS), version 9.4, will be used. All material used for data collection will be stored by the researcher for five years, remaining confidential. The results of the study will be published in an indexed scientific journal. This article has followed the SPIRIT guidelines for its elaboration [16].

## Discussion

Insertion of an IUD in the immediate postpartum period has been considered a good option to increase coverage and access to contraception, and its benefit outweighs the inconvenience of a higher expulsion rate. Studies show that it is safe but has higher expulsion rates [17–20].

The vast majority of studies used manual insertion of copper IUDs. Regarding the use of the modified Kelly placental forceps, there are few studies that assess its complications and the comparison between its use and manual insertion [15, 20]. Counseling women is difficult when evidence from randomized controlled trials is limited. The benefit of providing highly effective contraception immediately after delivery may outweigh the disadvantage of the increased risk of expulsion. It is not known whether the use of modified Kelly forceps for immediate postpartum vaginal delivery interferes with expulsion rates compared to manual insertion.

A study comparing both methods of insertion, as well as the degree of discomfort and pain of women undergoing the procedure, is needed. We expect forceps insertion to be a more comfortable alternative for women without labor analgesia, but better placement and a lower expulsion rate are expected with manual insertion by placing the IUD closer to the uterine fundus. Providing access to effective and long-term contraception during hospitalization for childbirth is a fundamental strategy in combating the epidemic of unplanned pregnancy [21].

A high rate of acceptance of the IUD is expected among the women we tend to because at the Women’s Hospital, there is constant training of professionals for insertion of the device, one of the main obstacles mentioned in the literature to the use of the method [22]. There is also a wide dissemination of the benefits of long-term reversible contraceptive methods during prenatal appointments. This is a different scenario from the rest of the country, in which only 2% of women of childbearing age have access to the IUD [7].

## Trial status

The study is in the participant recruitment phase. So far, 30 participants have been included. Recruitment of participants started in July 2022 and is expected to end in July 2023.

## Supplementary Information

**Additional file 1.**

**Additional file 2.** Questionnaire 1.

**Additional file 3.** Questionnaire 2.

## Data Availability

Not applicable.

## Abbreviations

IUD: Intrauterine device
UNICAMP: University of Campinas
REBEC: Brazilian Registry of Clinical Trials
SAS: Statistical Analysis System
CAAE: Certificate of Presentation of Ethical Appreciation

## References

[1] Conde-Agudelo A, Rosas-Bermudez A, Castaño F, Norton MH (2012). Effects of birth spacing on maternal, perinatal, infant, and child health: a systematic review of causal mechanisms. Stud Fam Plann.

[2] Conde-Agudelo A, Belizán JM, Lammers C (2005). Maternal-perinatal morbidity and mortality associated with adolescent pregnancy in Latin America: cross-sectional study. Am J Obstet Gynecol.

[3] DaVanzo J, Hale L, Razzaque A, Rahmand M (2007). Effects of interpregnancy interval and outcome of the preceding pregnancy on pregnancy outcomes in MATLAB, Bangladesh. BJOG.

[4] Cleland J, Bernstein S, Ezeh A, Faundes A, Glasier A, Innis J (2006). Family planning: the unfinished agenda. Lancet.

[5] Rossier C, Bradley SEK, Ross J, Winfrey W (2015). Reassessing unmet need for family planning in the postpartum period. Stud Fam Plann.

[6] Surita FGC, Luz AG, Hsu LPR, Carvalho FHC, Brock MF, Nakamura MU (2020). Outpatient care for pregnant and puerperal women during the COVID-19 pandemic. Rev Bras Ginecol Obstet.

[7] Barbieri MM, Herculano TB, Dantas Silva A, Bahamondes L, Juliato CR, Surita FG (2021). Acceptability of ENG-releasing subdermal implants among postpartum Brazilian young women during the COVID-19 pandemic. Int J Gynecol Obstet.

[8] Echeverry G (1973). Family planning in the immediate postpartum period. Stud Fam Plann.

[9] Jackson E, Glaiser A (2011). Return of ovulation and menses in postpartum nonlactating women: a systematic review. Obstet Gynecol..

[10] Sok C, Sanders JN, Salzman HM, Turok DK (2016). Sexual behavior, satisfaction, contraceptive use among postpartum women. J Midwifery Women Health.

[11] Marangoni M, Laporte M, Surita F, Kraft MB, Bahamondes L, Juliato CRT (2021). One-year follow up on postplacental IUD insertion: a randomized clinical trial. Acta Obstet Gynecol Scand.

[12] Kapp N, Curtis KM (2009). Intrauterine device insertion during the postpartum period: a systematic review. Contraception.

[13] Grimes DA, Lopez LM, Schulz KF, Van Vilet HAAM, Stanwood NL. Immediate postpartum insertion of intrauterine devices. Cochrane Database of Systematic Reviews. 2010;5:1-8.

[14] Hulley SB, Cummings SR, Browner WS, Grady DG, Newman TB (2007). Designing Clinical Research.

[15] Gurney EP, Sonalkar S, McAllister A, Sammel MD, Schreiber CA (2018). Six-month expulsion of postplacental copper intrauterine devices placed after vaginal delivery. Am J Obstet Gynecol.

[16] Chan AW, Tetzlaff JM, Altman DG, Laupacis A, Gøtzsche PC, Krleža-Jerić K (2013). SPIRIT 2013 statement: defining standard protocol items for clinical trials. Ann Intern Med.

[17] Averbach SH, Ermias Y, Jeng G, Curtis KM, Whiteman MK, Berry-Bibee E, Jamieson DJ, Marchbanks PA, Tepper NK, Jatlaoui TC (2020). Expulsion of intrauterine devices after postpartum placement by timing of placement, delivery type, and intrauterine device type: a systematic review and meta-analysis. Am J Obstet Gynecol.

[18] Makins A, Taghinejadi N, Sethi M, Machiyama K, Munganyizi P, Odongo E (2018). FIGO postpartum intrauterine device initiative: complication rates across six countries. Int J Gynecol Obstet.

[19] Singh S, Das V, Agarwal A, Dewan R, Mittal P, Bhamrah R, Lerma K, Blumenthal PD (2016). A dedicated postpartum intrauterine device inserter: pilot experience and proof of concept. Glob Health Sci Pract.

[20] Blumenthal PD, Lerma K, Bhamrah R, Singh S (2018). Comparative safety and efficacy of a dedicated postpartum IUD inserter versus forceps for immediate postpartum IUD insertion: a randomized trial. Contraception..

[21] Bahamondes L, Fernandes A, Monteiro I, Bahamondes MV (2020). Long-acting reversible contraceptive (LARCs) methods. Best Pract Res Clin Obstet Gynecol.

[22] Laporte M, Becerra A, Castro L (2021). Evaluation of clinical performance when intrauterine devices are inserted by different categories of healthcare professional. Int J Gynecol Obstet.

