# Supplementary material for: Comparison between two methods of the immediate post-placental insertion of copper intrauterine device in vaginal birth—a protocol for a randomized clinical trial
Source: Trials. 2022 Dec 27;23:1053. doi: 10.1186/s13063-022-07041-x (PMC9793389; doi:10.1186/s13063-022-07041-x)
Supplement: Supplementary file 2 — Additional file 2. Questionnaire 1. [file 13063_2022_7041_MOESM2_ESM.docx]

**“COMPARISON BETWEEN TWO METHODS OF THE IMMEDIATE POST-PLACENTAL INSERTION OF COPPER INTRAUTERINE DEVICE IN VAGINAL BIRTH”**

Date: ___/___/_____ Number:|__|__|__| __|

Initials: |__|__|__|__|__|__|__| Medical record: [__|__|__|__|__|__|__|__|__]

1. Birth date: ___/___/_____ 2. Age: ________

3. Skin color: White [ 1 ] Black [ 2 ] Brown [ 3 ] Yellow [ 4 ] Indigenous [ 5 ]

4. Marital status: With partner [ 1 ] No partner [ 2 ]

5. Student: Yes [ 1 ] No [ 2 ] 6. Scholarity: ________________________

7. Profession:_____________________ 8 Wage: ___________.

9. Pregnancies _____ Births_______.

10. Gestational age: __________

**11. Reasons for acceptance:** _____________________________________________________

**12. Desire for a new pregnancy: Yes [ ] No [ ]**

**13. Insert type: Manual [ ] Clamp[ ]**

**14. Discontinuation before insertion: Yes [ ] No [ ]**

**If yes, Reason:**

[ ] Fever during labor or delivery

[ ] Amniorrhexis for more than 24 hours

[ ] Manual placental extraction

[ ] Postpartum hemorrhage or uterine atony

**15. Type of delivery**: **Vaginal [ ] Forceps** **[ ] 16. Analgesia: Yes [ ] No [ ]**

**17. Episiotomy: Yes [ ] No [ ]**

**18. Laceration: Yes [ ] No [ ] If yes, Grade: I [ ] II [ ] III[ ] IV[ ]**

**19. pain during insertion (0-10): _______ 20. Insertion difficulty (0-10): _______**
