# Supplementary material for: Comparison between two methods of the immediate post-placental insertion of copper intrauterine device in vaginal birth—a protocol for a randomized clinical trial
Source: Trials. 2022 Dec 27;23:1053. doi: 10.1186/s13063-022-07041-x (PMC9793389; doi:10.1186/s13063-022-07041-x)
Supplement: Supplementary file 3 — Additional file 3. Questionnaire 2. [file 13063_2022_7041_MOESM3_ESM.docx]

**“COMPARISON BETWEEN TWO METHODS OF THE IMMEDIATE POST-PLACENTAL INSERTION OF COPPER INTRAUTERINE DEVICE IN VAGINAL BIRTH”**

Date: ___/___/_____ Number:|__|__|__| __|

Initials: |__|__|__|__|__|__|__| Medical record: [__|__|__|__|__|__|__|__|__]

- Are you using the IUD?: Yes [ 1 ] No [ 2 ]
- Expelled the IUD? Yes [ 1 ] No [ 2 ] – Date:___/____/_____
- Is pregnant? Yes [ 1 ] No [ 2 ]
- Need for removals whether for pain, bleeding, personal reasons or other medical reasons: Yes [ 1 ] No [ 2 ]
- If yes, which one?:__________________
- Uterine perforation Yes [ 1 ] No [ 2 ]
- Infection Yes [ 1 ] No [ 2 ]
- Menstrual pattern:

[ ] Amenorrhea [ ] Infrequent bleeding

[ ] Frequent bleeding [ ] Irregular bleeding

- Breastfeeding

[ ] Exclusive [ ] Predominant [ ] Mixed

[ ] Stopped breastfeeding

- IUD placement on ultrasound: [ ] adequate [ ] misplaced [ ] unviewed
- Do you want to continue using the IUD? Yes [ 1 ] No [ 2 ]

– Reason: ­­­­­­­­­­­­­­­­­­________________________________

- If not, which contraceptive method do you want?:____________________

- Satisfaction with the IUD

1. Are you satisfied with the IUD you are using?

| 1 | 2 | 3 | 4 | 5 | 6 | 7 | 8 | 9 | 10 |
| --- | --- | --- | --- | --- | --- | --- | --- | --- | --- |

1. Would you recommend the IUD to other people?

| 1 | 2 | 3 | 4 | 5 | 6 | 7 | 8 | 9 | 10 |
| --- | --- | --- | --- | --- | --- | --- | --- | --- | --- |
